# The use of missing values in proteomic data-independent acquisition mass spectrometry to enable disease activity discrimination

**DOI:** 10.1093/bioinformatics/btz898

**Published:** 2019-12-02

**Authors:** Kathryn A McGurk, Arianna Dagliati, Davide Chiasserini, Dave Lee, Darren Plant, Ivona Baricevic-Jones, Janet Kelsall, Rachael Eineman, Rachel Reed, Bethany Geary, Richard D Unwin, Anna Nicolaou, Bernard D Keavney, Anne Barton, Anthony D Whetton, Nophar Geifman

**Affiliations:** 1 Division of Cardiovascular Sciences, School of Medical Sciences, Faculty of Biology Medicine and Health, University of Manchester, Manchester, UK; 2 Stoller Biomarker Discovery Centre, Division of Cancer Sciences, School of Medical Sciences, Faculty of Biology, Medicine and Health, University of Manchester, Manchester Academic Health Science Centre, Manchester, UK; 3 Laboratory for Lipidomics and Lipid Biology, Division of Pharmacy and Optometry, UK; 4 Division of Informatics, Imaging and Data Sciences, School of Health Sciences, Faculty of Biology, Medicine and Health, University of Manchester, UK; 5 NIHR Manchester Biomedical Research Centre, Manchester Academic Health Science Centre, Manchester University NHS Foundation Trust, Manchester, UK; 6 Arthritis Research UK Centre for Genetics and Genomics, Centre for Musculoskeletal Research, University of Manchester, Manchester, UK

## Abstract

**Motivation:**

Data-independent acquisition mass spectrometry allows for comprehensive peptide detection and relative quantification than standard data-dependent approaches. While less prone to missing values, these still exist. Current approaches for handling the so-called missingness have challenges. We hypothesized that non-random missingness is a useful biological measure and demonstrate the importance of analysing missingness for proteomic discovery within a longitudinal study of disease activity.

**Results:**

The magnitude of missingness did not correlate with mean peptide concentration. The magnitude of missingness for each protein strongly correlated between collection time points (baseline, 3 months, 6 months; *R* = 0.95–0.97, confidence interval = 0.94–0.97) indicating little time-dependent effect. This allowed for the identification of proteins with outlier levels of missingness that differentiate between the patient groups characterized by different patterns of disease activity. The association of these proteins with disease activity was confirmed by machine learning techniques. Our novel approach complements analyses on complete observations and other missing value strategies in biomarker prediction of disease activity.

**Supplementary information:**

Supplementary data are available at *Bioinformatics* online.

## 1 Introduction

Proteomic biomarkers have a range of uses in respect to prediction of disease risk, response to therapy, prognosis and diagnosis. The discovery of proteomic biomarkers for these purposes can enable better patient stratification and disease management. Large biobank studies have been created, including UK Biobank (Sudlow *et al.*, 2015) and 100 000 Genomes Project (Caulfield *et al.*, 2017), which emphasize the interrogation of large-scale cohorts to uncover the cause of disease progression. Such biobanks create a fertile ground for discovery of new biomarkers using the many samples collected. In clinical research, over 200 specific proteins are measured to guide clinical decisions, and new interdisciplinary omics analyses will enable development of novel approaches to patient stratification and health care. As an example, in the study of cancers, measurement of circulating tumour DNA and proteins has enabled risk detection algorithms to be developed (Cohen *et al.*, 2018). Thus, to achieve this high demand, there is a necessity for high sample throughput, specificity, quantitative reproducibility and deep coverage proteomics techniques to profile molecular signatures that report on the state of the patient at a given time and relate this to a genomic propensity to a condition; risk of ill health or potential (adverse) response to treatment.

Sequential window acquisition of all theoretical mass spectra (SWATH) is a data-independent acquisition mass spectrometry technique created to be a reproducible, label-free method of proteomic analyses, with the sensitivity of targeted methods but with increased proteome depth (Gillet *et al.*, 2012). The use of cyclical acquisition covering the entire m/z range allows for retrospective interrogation of all peptides in a sample with the use of peptide reference libraries. SWATH is more reproducible in peptide identification and identifies more peptides than data-dependent methods (Krasny *et al.*, 2018), therefore, the resulting data has fewer missing values although it offers informatic challenges.

The handling of data containing missing values in biomarker discovery and proteomics remains a challenge and is not yet standardized (Lazar *et al.*, 2016; Webb-Robertson *et al.*, 2015; Wei *et al.*, 2018). Missing values in a mass spectrometry setting can be due to variation at a number of sources in the analysis pipeline from sample storage, extraction, losses in protein digestion and separation, measurement failure during mass spectrometry acquisition, relative quantification and depends on the quality control thresholds used and the signal-to-noise ratio. Missingness has been generalized into three types (Rubin, 1976), missing completely at random, missing at random and missing not at random, which are usually handled by imputation approaches specific to the type of missingness (Lazar *et al.*, 2016). For the handling of values missing not at random, the limit of detection (LOD) of a mass spectrometer is usually the focus of missingness approaches. Data are produced with missing values of this sort are ‘left-censored’ data where the distribution of the proteins’ abundance is truncated on the left side, due to the lower abundances not detected by the mass spectrometer at the LOD (Lazar *et al.*, 2016).

Missing values cannot be analysed by standard statistical tests, which has led to three principle methods of approaching missing data:

1. Devoting laboratory time and money to reprocessing and rerunning samples.
2. Removing the participants, samples or analytes with most missing values from statistical analyses, and therefore, diminishing the scope of the study due to the loss of data and study power.
3. Imputation via the replacement of missing values found in the raw data with one number, zero, a small value, the mean (Gromski *et al.*, 2014) or variations of calculations around the value of the LOD (Hornung and Reed, 1990); any of these approaches may introduce selection biases in the distributions of the proteins. Alternatively, imputation techniques to create a varying value for each missing value present through correlations or other statistical estimations may be used (Oba *et al.*, 2003).

There are many imputation approaches and software tools now available for mass spectrometry (Choi *et al.*, 2019; Karpievitch *et al.*, 2012; Wei *et al.*, 2018), however, there are many cases where imputation is not accurate enough in metrics of variance and sample classification (Webb-Robertson *et al.*, 2015); for example, if there is a high level of missing data, if the sample size is small, if a large subset of analytes have at least one missing value at random and the training set, therefore, contains a small number of complete observations, or when the ratio between the number of values used for the training set is small compared to the number requiring imputation from that training set. Therefore, there is a need to analyse missing values as they are found in the raw data, to complement the current analyses of observed data in biomarker discovery.

In metabolomics and subsequent biomarker studies, the handling of missing data is often by the ‘80% rule’ (Yang *et al.*, 2015), which is a threshold for complete observations, keeping only those metabolites identified in at least 80% of samples. This threshold is arbitrary and may not be appropriate for studies including longitudinal data such as drug treatment response studies, where such a threshold likely leads to the loss of potentially interesting data. There is no agreed set threshold specifically for proteomics studies.

The coverage of the proteome achieved by undertaking SWATH allows for the analysis of a novel subgroup of missing not at random values: missing with interest or biologically missing. As SWATH technology allows for more consistency and reproducibility in the analyses of the proteome, we present a novel approach of analysing missing values in a longitudinal study for the discovery of biomarkers associated with disease activity; an example of a study setting where missing values can be informative and such analyses complement current measurement protocols. We highlight the value of missing values in deep proteomic biomarker studies.

## 2 Materials and methods

### 2.1 Participants and samples

Rheumatoid arthritis patients were recruited at baseline and were assessed at follow-up at 3 and 6 months. About 64 participants were selected for SWATH proteomic analyses according to their disease activity and used for the informatics study paradigm described below. Serum samples were collected from the 64 participants over the three time points; 58 participant samples were measured at baseline, 47 at 3 months and 44 at 6 months. Disease activity at 6 months was scored using an algorithm derived to combine data on standard markers of the disease, and this outcome was categorized into low- and high disease activity using established criteria (Prevoo *et al.*, 1995), which was used as the outcome for all analyses (Supplementary Methods). From the 64 participants selected for proteomic analyses, 32 participants had low disease activity, 12 had high disease activity and 20 had low disease activity at 3 months but progressive worsening by 6 months (secondary high disease activity group). The study complies with the declaration of Helsinki. Participants provided written informed consent, and the study is ethically approved (COREC 04/Q1403/37). The proteomics methodology can be found in the Supplementary Methods.

### 2.2 Post-acquisition bioinformatics and relative quantification

The SWATH map from the mass spectrometer was converted from proprietary SCIEX format (.wiff and accompanying scan files) to .mzML open-format for the OpenSWATH engine using the SCIEX MS Data Converter (Beta 1.3) under profile mode. The resulting .mzML files were time-normalized against an iRT library and interrogated with a serum-focused spectral library (Röst *et al.*, 2014) using the OpenSWATH single executable (OpenSWATHWorkflow version 2.0.1). Parameters were set as previously described (Navarro *et al.*, 2016). The OpenSWATH output files were subjected to pyProphet algorithm (Teleman *et al.*, 2015) for false discovery assessment of the inferred transition groups. The outputs were aligned using the TRIC algorithm (Röst *et al.*, 2016), to retain only consistent inferred transition groups. Proteotypic peptides were quantotypic (criterion of one peptide per protein) with a 15% consensus filter, or appear in both duplicate injections. Quality control filtering included removal of sample data if the coefficient of variation across duplicates was above a median of 20%. MSstats from Bioconductor in R (Choi *et al.*, 2014) was used for normalization and protein quantification. The equalize medians and Top 3 features per peptide options parameters were used.

### 2.3 Statistical analyses

#### 2.3.1 Missing value threshold

Proteins that were not detected in >10% of total samples were removed from the analyses (required > 15 observations of the total 149). This threshold was used after assessment of the distribution of total missingness, which showed an inflated distribution of proteins with <15 observations (see Supplementary Figs S1 and S2). The removal of the proteins with <10% total observations ensures that proteomic outliers in specific discrete samples were excluded from analyses, which may arise from individual heterogeneity in this small cohort or underlying mass spectrometry noise, which is not biologically informative. Therefore, acceptance of proteins that are present in at least 10% of measurements confirms the mass spectrometry reproducibility in multiple disease activity groups at different time points. Thus, the 565 proteins that remained from the initially identified 742 that were used for further analyses are unlikely to be outliers and their presence has been reproducibly confirmed by the mass spectrometer and further quality control assessments. R software (version 3.5.2) was used for all analyses and graphic plotting.

#### 2.3.2 Assessment of proteomic outliers via magnitude of missingness

We hypothesized that the most informative proteins would be reproducibly missing in a similar pattern for all patients with the same disease activity, regardless of the time point of collection (of which there are three); such proteins would be consistently missing/present over the three time points. To assess if each protein’s presence or missingness was similar over time, regardless of disease status, the strength of relationship between time points was calculated via Pearson’s correlation coefficient by counting the missing values for each protein, at each of the three time points separately, and adjusting by the number of samples collected at that time point (Equation 1). To identify proteins whose presence or absence altered with disease activity, the strength of relationship was assessed by counting missing values for each protein, in each of the three disease activity statuses, regardless of time point, and adjusting for the number of samples collected for certain disease activity (Equation 1). The relationship of a protein's missingness was plotted and outliers were visually identified for further assessment. The magnitude of missing values (α) used for all missing value analyses is described by the count of missing values normalized by the number of samples at each time point or disease activity group, as depicted in Equation 1 (magnitude of missingness).

(1) $$\frac{\mathrm{Count}\;\mathrm{of}\;\mathrm{missing}\;\mathrm{values}}{\mathrm{Number}\;\mathrm{of}\;\mathrm{samples}}=\mathrm{Normalized}\;\mathrm{missing}\;\mathrm{count}=\alpha$$

#### 2.3.3 Machine learning protocol

Feature selection and machine learning were undertaken using the framework proposed by Perez-Riverol *et al.* (2017), to identify proteins at baseline and at 3 months that predict the 6-month disease activity outcome. Feature selection removed proteins by univariate correlation of *R* < 0.3 with disease activity, and by multivariate correlation of *R* > 0.75 with each other. Recursive feature elimination and importance evaluation identified the best protein predictors of the disease outcome via Random Forest. The missing values for the comparative machine learning analyses were set to zero, as the exploited approach does not handle missing values.

#### 2.3.4 Assessment of batch effects

Assessment of batch effects on the magnitude of missing values was completed in four ways:

- 1. $\frac{\alpha }{\mathrm{Sum}\;\mathrm{of}\;\mathrm{batches}\;(1-12)}$
- 2. $\frac{\alpha }{\mathrm{Frequency}*\mathrm{Count}\;\mathrm{of}\;\mathrm{batches}\;(1-12)}$
- 3. $\frac{\mathrm{Count}\;\mathrm{of}\;\mathrm{missing}\;\mathrm{values}\;(\mathrm{unnormalized})}{\;\mathrm{Frequency}*\mathrm{Count}\;\mathrm{of}\;\mathrm{batches}\;(1-12)}$
- 4. $\frac{\alpha }{\mathrm{Count}\;\mathrm{of}\;\mathrm{batches}\;(1-12)}$

## 3. Results

### 3.1 Participant characteristics

Serum samples of 64 participants from three time points were analysed. Table 1 highlights the description of the participants included. There was little correlation between age [*R* = −0.18, confidence interval (CI) = −0.41 to 0.07] or gender (*R* = −0.08, CI = −0.32 to 0.17) and disease activity group (distributions depicted in Supplementary Fig. S3).

**Table 1.** Summary statistics of the study participants

Trait	Mean (range)		
DA	High	2° High	Low
*N*	12	20	32
Age (years)	63 (21–82)	66 (37–81)	59 (28–81)
Gender	8% male	20% male	19% male
BMI^a^	32.29 (22.27–50.32)	26.61 (17.04–35.80)	29.43 (19.71–43.70)

*Note*: The mean and range for each trait that describes the 64 participants included in the proteomic analyses are shown.

BMI, body mass index; DA, disease activity; *n*, sample size.

a BMI is measured at baseline.

### 3.2 SWATH missingness is not due to LOD

A total of 565 proteins were included in the analysis; 99 proteins had complete observations in all serum samples, the remainder of proteins contained at least one missing value (Supplementary Table S1). We addressed the hypothesis that missing values found in SWATH analyses are increased in low abundance proteins where concentrations are near or below the LOD of the mass spectrometer. We found a weak relationship between the mean log abundance of the proteins and the count of missing values in the 149 samples (*R* = −0.37, CI = −0.44 to −0.30, Supplementary Fig. S1), as well as when the samples are separated by disease activity group and collection time point (*R* = −0.33 (CI = −0.40 to −0.25) to *R* = −0.09 (CI = −0.17 to −0.01), Supplementary Fig. S2). We concluded that when using the SWATH mass spectrometry methodology, including the standard bioinformatic approaches, missingness is not strictly left-censored and this suggests that missingness might be reproducible and informative at the biological level. Such missing values could, therefore, be treated as measures in themselves, rather than due to methodological issues in measuring them (e.g. mass spectrometry matrix effects).

### 3.3 Proteomic missingness is similar over time

**Fig. 1.**
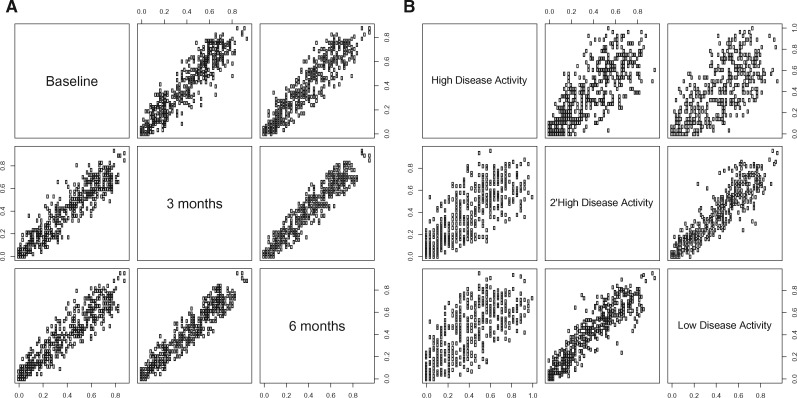
The assessment of missingness for each protein over collection time points and by disease activity status. (**A**) The correlation between protein missingness over three time points showed a strong relationship; the magnitude of missingness for each protein measured at baseline correlated with those measured at 3 months and with those at 6 months to *R* = 0.95 (CI = 0.94–0.96). The protein missingness measured at 3 months correlated with those measured at 6 months to *R* = 0.97 (CI = 0.96–0.97). (**B**) The correlation between protein missingness separated by response status to treatment. The magnitude of missingness for each protein identified in the high disease activity group correlated with those measured in secondary high disease activity group to *R* = 0.84 (CI = 0.82–0.86), and to those measured in low disease activity group to *R* = 0.82 (CI = 0.79–0.84). The protein missingness measured in secondary high disease activity group correlated with that of low disease activity group to *R* = 0.94 (CI = 0.93–0.95)

Since it is possible that some protein levels alter markedly over the time course of the study, we examined how levels of missingness change over time. There was higher magnitude of missingness (see Equation 1) in proteins measured at the baseline collection of samples (58 samples, 39% missing protein values) than the samples collected at 3 months (47 samples, 31% protein missing values) or 6 months (44 samples, 30% protein missing values). We assessed the strength of the relationship of magnitudes of missingness for each protein between the time points (Fig. 1). This relationship was strong (*R* = 0.95–0.97, CI = 0.94–0.97), showing that the magnitude of missingness for each protein was similar regardless of time point of collection. This similarity in missingness is likely due to the reproducibility of the technique; identifying proteins in the same way in each sample, or further, from the fidelity of the bioinformatics workflow; assessing the certainty of the presence of the peptide with appropriate quality control filters. This reproducibility is a firm foundation for the analyses of missing data, as any deviations in missingness are more likely due to a biological change.

### 3.4 Proteomic missingness differs by disease activity

The relationship strength and similarity found previously in the analysis of the magnitude of missingness between time points, decreased when comparing between disease activity groups. Figure 1 also depicts the correlation between the disease activity groups for the magnitude of missingness of each protein. Missingness in the high disease activity group correlated with secondary high disease activity to *R* = 0.84 (CI = 0.82–0.86), and with low disease activity to *R* = 0.82 (CI = 0.79–0.84). Such strength of relationship was stronger between the missingness of peptides between secondary high disease activity and low disease activity, at *R* = 0.94 (CI = 0.93–0.95), which is likely due to the secondary high disease activity participants having a favourable disease activity until the 3-month time point. As the strength of relationship between missingness at the different time points was *R* > 0.95, and the strength of relationship when analysing the magnitude of protein missingness by the disease activity of the participant was lower (*R* < 0.94), outliers of missingness can be assessed from correlation with disease activity status, and such outliers may be informative of a biological change due to the response to treatment.

**Fig. 2.**
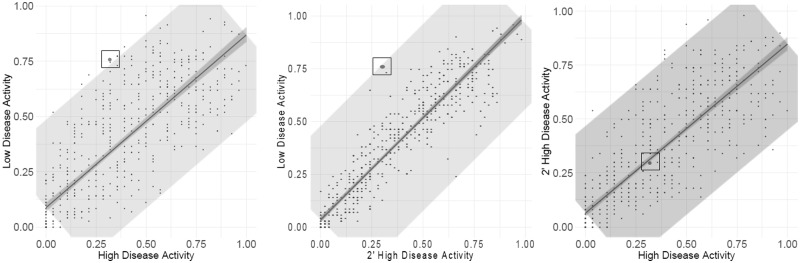
Identification of an example outlier protein (in the square) as a predictor of disease activity from the assessment of missing values. The outlier protein is identified as an outlier due to increased missingness count in low disease activity participants when compared to both types of high disease activity groups. The protein’s missingness does not separate those at particularly high disease activity participants from secondary high disease activity participants. The shaded area is a line parallel to the linear regression line, expanded in size

In this analysis outliers of missingness were identified; one of these is highlighted in Figure 2. An outlier when comparing the magnitude of missingness between low disease activity and both types of high disease groups was found, demonstrating a higher than expected degree of missingness in the low disease activity group. This suggests that the protein may predict the underlying biology when comparing low disease activity with any high disease activity category and offers encouragement that this approach can identify biomarkers to be validated further. The protein did not demonstrate outlier behaviour in the correlation of magnitude of missingness between high disease activity and secondary high disease activity, and therefore, does not differentiate between these two groups. Further, it was not found to be an outlier of missingness when comparing the different time points (Supplementary Fig. S4). This example demonstrates how correlation analysis assessing the relationship of total missingness in all measurements, identified a protein that can potentially discriminate in respect of disease activity status.

### 3.5 Missingness outliers may associate with disease activity

Further descriptions of the protein identified as an outlier of missingness between response groups are shown in Table 2. The table shows the raised level of missing values identified for the measurement of this protein in low disease activity participants at each time point. The protein is a candidate biomarker of high disease activity due to its raised number of observations. To assess how abundant this protein is usually in serum, we used the Protein Abundance Database, PaxDb (Wang *et al.*, 2015), a comprehensive absolute protein abundance database. In humans, the protein is very abundant in multiple cells and tissues around the body, while there is no data on serum expression; it is found at lowest identifiable abundance in plasma (0.04–0.11 ppm range). This may suggests that an abnormal increased concentration can be indicative of disease pathology. This, and other identified outlier proteins were not found to be enriched with any specific protein domains or functional categories, as assessed by enrichment analysis (Supplementary Methods).

**Table 2.** Missingness summary statistics of an outlier protein

Time	*N*	HD	2°HD	LD	*n* (miss)	% (miss) in LD
Baseline	58	5 (42%)	9 (47%)	21 (77%)	35	60%
3 months	47	0 (0%)	4 (27%)	18 (78%)	22	82%
6 months	44	4 (57%)	2 (13%)	15 (71%)	21	71%
Total	149	9 (32%)	15 (30%)	54 (76%)	78	69%

*Note*: High disease participants (HD), secondary high disease participants (2°HD), and low disease participants (LD) are described. HD and 2°HD had low levels of missingness at each time point of collection, while LD showed >2-fold levels of missingness at all time points. n = count of participants at each time point; % indicates missing values of the total participants with that outcome at that time point; n(miss) = the count of total missing values at each time point; and %(miss) in LD is the % of total missingness for the outlier protein found in LD at each collection time point. The total contains a sum of the counts and the mean % in brackets.

### 3.6 Confirmation of a missingness outlier protein as a predictor of disease activity by machine learning techniques

Clearly, our data also contain relative quantitation measurements of the outlier protein in the samples where it was detected. Machine learning was used as previously published (Perez-Riverol *et al.*, 2017) to identify proteomic biomarkers that predict the disease activity at 6 months based on the measured levels within the same analysis.

Using the observed values for the proteomic biomarkers measured at baseline (as opposed to magnitude of missingness), Random Forest identified the exemplar outlier protein alongside 21 other proteins in the prediction of disease activity (Supplementary Fig. S5). Using the proteomic biomarkers measured at the 3-month time point, the outlier protein was identified with only three other proteins as predictors of disease activity (Supplementary Fig. S6), supporting our identification of this protein from our analysis of missingness.

### 3.7 Batch does not affect the missingness results

The samples were run together in the proteomic analyses, i.e. each participant’s samples (baseline, 3 months, 6 months) were run sequentially. We, therefore, assessed the effect of batch on the missingness outcomes of disease activity status. There were 12 batches used to run the samples, Supplementary Figure S7 depicts the distributions of the samples at each time point and disease activity status over the batches. Samples of all time points were distributed over all 12 batches. High disease activity samples were distributed over only two batches, secondary high disease activity samples over eight batches and low disease activity samples over 10 batches. All four adjustments to assess batch effects (see Section 2.3.4 ) did not affect the results, including the relationships of missingness or on the outlier protein (Supplementary Fig. S8).

### 3.8 Reproducible missingness is created by standard SWATH bioinformatic approaches

The bioinformatics tools align the samples to retain only consistently inferred transition groups. At this point, the presence of any peptide for the exemplar protein was investigated in the samples to explore where the missing values were created along the bioinformatic pipeline (Supplementary Fig. S9). As expected, there was an increase in the magnitude of missing values for the protein after bioinformatic quality control thresholds were applied (post pyProphet, TRIC and thresholds applied). However, the trend of missing values between collection time points was comparable before and after bioinformatic quality control. The missing values were assessed similarly against disease activity, which showed that the low disease activity participants had an increased magnitude of missing values both before and after bioinformatic quality control when compared with high disease activity participants. This highlights the biological importance of this protein, which was more likely missing in low disease activity participants and more identifiable in high disease activity participants from the mass spectrometry data. The missingness is increased by the bioinformatic quality control in the identification of high-fidelity assignment of protein identity using standard proteomic approaches.

### 3.9 Further analyses of current missingness thresholds

The effect of specific thresholds in our biomarker discovery process was assessed; three based on the percentage of missing values (<90%, <50% and <20%), and one using a discrete value based on the distribution of missingness, which showed a dip in the frequency of proteins with <50 missing values (see Supplementary Fig. S10). The same machine learning technique was used to identify proteomic biomarkers that predict disease activity using each of the different missingness thresholds. The best performance was achieved for proteins at baseline and 3 months when using a threshold of >10% observations (<90% missing values) (Supplementary Fig. S11). This accuracy is less due to the differing number of proteins included at each threshold, as the correlation between the number of proteins and the accuracy of the machine learning was *R* = 0.48 (CI = −0.21 to 0.85; *n* = 10), but more likely the removal of noise and the underlying assumption by the machine learning software that the proteins with the most missing values are lowest due to the zero imputation. Further correlation plots and Principal component analyses using the different missing values thresholds are shown in Supplementary Figure S12, where the strength of relationship between the missingness and time point or disease activity status decreases with the missing values lost at each observation threshold.

## 4 Discussion and conclusions

We present an alternative approach for the analysis of missing data in a SWATH mass spectrometry setting for an exemplar set of 149 samples. This complements the analyses of complete observations, imputation, and other missing value replacement statistical techniques. We show that such analyses allow for the identification of proteins that differ in missingness by disease activity. We confirmed the predictive importance of an exemplar protein by machine learning techniques. Similarly, we show that this protein was observed more in high disease activity participants, with more missingness found in patients with low disease activity.

The 90% missingness threshold (10% observations), which includes any biomarker found with at most 90% missing values, allowed for better accuracy in the prediction of proteomic biomarkers of disease activity and therefore showed that more stringent thresholds decrease prediction accuracy and remove potentially important discoveries. As there was a weak relationship between the number of proteins included in the analysis and the accuracy of the analysis, this high accuracy found when using the 90% missing threshold is likely due to the importance of missing values on prediction of disease activity. Had a missing value threshold of 80% been used (maximum 20% missing values), as is usual of mass spectrometry studies, the outlier protein we identified as a marker of low activity would have been removed from analyses and left undiscovered.

Missingness as an informative tool is not a novel concept; missingness is informative when a value is missing due to a test not being carried out because of a reason that is not missing at random. For example, this can be due to the test being left unanswered; where personal information is involved, where it was answered as ‘Prefer not to answer’, where it is thought that scoring highly is unlikely, or where a test is only provided to subgroups of participants (e.g. medical screening specific for certain genders, ages, risk populations, or disease activity). For example, a portion of the UK female population do not have cervical smear test results, as many do not attend their free testing appointment (77% attendance rate). Some of the reasons provided for non-attendance include embarrassment, negative perceptions of health professionals, worry and trust in results, concerns about the procedure, idiosyncratic beliefs, extreme negative experiences and not receiving prompts to be screened (Marlow *et al.*, 2019). In some cases, the absence of data can be informative by its own accord, as in electronic health records (EHRs). Missing values in this setting may be due to biased screening, tests only conducted if clinically relevant to specific ailments, or information being fragmented across multiple healthcare systems (Beaulieu-Jones *et al.*, 2018). Thus, in some scenarios, no test result can potentially be informative. While it may be possible that missingness is not informative in such a way in a mass spectrometry setting, informative missingness can be exampled by blood antibody rheumatoid factor, where the concentration correlates with severity of rheumatoid arthritis. This test is typically only performed for patients with some indication of rheumatoid arthritis. Thus, patients with high rheumatoid factor levels are more likely to have rheumatoid factor measures, while the missing values could not be imputed (Mason *et al.*, 2010).

The need for novel biomarkers of disease has led to an increase in the collection of large-scale cohorts and consortiums, in the aim of identifying biological predictors that stratify patients with polymorphic phenotypes, differing response to treatment, or those at risk of the occurrence of a disease event. Many cohorts have limited samples and resources; it is of great importance that the full benefit of the effort dedicated to the cohort collection and funding, is achieved in the resulting discoveries. SWATH-MS is a particularly useful tool for large-scale proteomics due to its reproducibility, analysis depth and the ability to recursive data mine for proteomic biomarkers of interest via differential peptide/protein reference libraries. The proteomic coverage obtained from SWATH results in the production of fewer missing values and we show here that they are not fully due to the LOD. The proteins that are of an average log intensity but identified in few samples are likely due to the quality control thresholds of the retrospective data interrogation used for SWATH proteomics (Fu *et al.*, 2018). As the assumption of missing-at-random (MAR) cannot be verified from observed data, sensitivity analyses have been used to assess the impact on results of departures from the assumption of MAR (Leacy *et al.*, 2017). Here, we provide an additional method of such assessment for SWATH-MS data.

The generalisability of this approach to other datasets, which contain their own missing data patterns and processes, is currently unclear. However, the strength of the dataset used here is the availability of protein measurements at multiple time points, allowing for confirmation of reproducible missingness. Similarly, other application would also require similar data that allow the consistency of missingness to be tested. While not tested here, it is likely that this method can be applied to other analysis platforms, sample types and diseases, as it is the dataset of multiple time points and disease statuses that is of importance to the methodology. This is demonstrated by a similar study assessing mortality outcomes and ICD-9 criteria (Che *et al.*, 2018). As far as we are aware, there is no reason wider application could not be pursued. A limitation of this study is the relatively small sample size, and large cohort studies will aid this effort. In order to take forward any protein as a biomarker, the results will need to be validated in an independent cohort. Nevertheless, here, we demonstrate the proposed technique has potential and can contribute to presently employed informatics techniques. In conclusion, we have shown that the peculiar features of SWATH-MS allow for the inclusion of samples with missing values in statistical analysis for biomarker discovery. Furthermore, we have shown the usefulness of analysing missingness as it is found in raw data, complementing and expanding on the findings achievable with current missing value strategies.

## Funding

This work was supported by the Medical Research Council, UK through an MRC Doctoral Award and an MRC Flexible Training Supplement [MR/K501311/1 to K.M.]; and a University of Manchester President’s Doctoral Scholarship [to K.M.]. This work was further supported by the Medical Research Council and the Engineering and Physical Sciences Research Council [MR/N00583X/1 ‘Manchester Molecular Pathology Innovation Centre (MMPathIC): bridging the gap between biomarker discovery and health and wealth’ to A.D.W, N.G. and A.B.]; National Institute for Health Research Manchester Biomedical Research Centre [to D.P., A.B. and A.D.W.], Versus Arthritis [21754] and Bloodwise [13005]. Equipment at the Stoller Biomarker Discovery Centre is funded by the Medical Research Council [MR/M008959/1]. This work was also supported by the Cancer Research UK Manchester Centre award [C5759/A25254 to D.C. and A.D.W.]. B.D.K. is supported by a British Heart Foundation Personal Chair.

*Conflict of Interest*: none declared.

## Supplementary Material

btz898_Supplementary_Files
