# Supplementary material for: The use of missing values in proteomic data-independent acquisition mass spectrometry to enable disease activity discrimination
Source: Bioinformatics. 2019 Dec 2;36(7):2217–23. doi: 10.1093/bioinformatics/btz898 (PMC7141869; doi:10.1093/bioinformatics/btz898)
Supplement: btz898_Supplementary_Files [file btz898_supplementary_files.docx]

Supplementary Information

The use of missing values in proteomic data-independent acquisition mass spectrometry to enable disease activity discrimination

Kathryn A. McGurk^1,2,3^, Arianna Dagliati^4^, Davide Chiasserini^2^, Dave Lee^2^, Darren Plant^5^, Ivona Baricevic-Jones^2^, Janet Kelsall^2^, Rachael Eineman^2^, Rachel Reed^2^, Bethany Geary^2^, Richard D. Unwin^1,2^, Anna Nicolaou^3^, Bernard D. Keavney^1^, Anne Barton^5,6^, Anthony D. Whetton^2^, Nophar Geifman^4,^*

^1^Division of Cardiovascular Sciences, School of Medical Sciences, Faculty of Biology Medicine and Health, University of Manchester, Manchester, UK. ^2^Stoller Biomarker Discovery Centre, Division of Cancer Sciences, School of Medical Sciences, Faculty of Biology, Medicine and Health, University of Manchester, Manchester Academic Health Science Centre, Manchester, UK. ^3^Laboratory for Lipidomics and Lipid Biology, Division of Pharmacy and Optometry, Faculty of Biology Medicine and Health, University of Manchester, UK. ^4^Division of Informatics, Imaging and Data Sciences, School of Health Sciences, Faculty of Biology, Medicine and Health, University of Manchester, Manchester, UK. ^5^NIHR Manchester Biomedical Research Centre, Manchester University NHS Foundation Trust, Manchester Academic Health Science Centre, Manchester, UK. ^6^Arthritis Research UK Centre for Genetics and Genomics, Centre for Musculoskeletal Research, University of Manchester, Manchester, UK.

**Supplementary Figures**

**Figure S1**. The distribution of missing values over 149 samples from 64 participants over 3 time points. The figure depicts the distribution of the count of missing values for the 742 proteins identified. A) The count of missing values plotted against the mean log abundance. The correlation between protein missingness and mean value was R=-0.37. B) The distribution of missing values over 149 samples.

**Figure S2**. The distribution of missing values over 149 samples separated by time point and disease activity status. The plots are based on the measurement of proteins from 64 participants of three response groups, collected over 3 time points (baseline, 3-months, 6-months). The correlation between mean protein abundance and it’s level of missingness was R = -0.33 to -0.09.

**Figure S3.** Distribution of gender in disease activity groups. The cohort was 17% male. A) The number of men in each disease activity group. B) The number of women in each disease activity group. C-E) The count of women and men in each disease activity group.

**Figure S4.** The outlier protein identified from correlation against disease activity status is not an outlier of missingness when comparing collection time points (baseline, 3-months, 6-months).

**Figure S5.** Top two principal components are shown in a PCA plot which depicts the prediction of participant disease activity by proteomic biomarkers at baseline. 22 proteomic biomarkers predicted disease activity, including the exemplar missingness outlier protein, but the prediction did not separate the disease activity groups to a substantial extent. The class groups are coded high disease activity (class 0), secondary high disease activity (class 1), and low disease activity (class 2).

**Figure S6.** Top two principal components are shown in a PCA plot which depicts the prediction of participant disease activity by proteomic biomarkers at the 3-month time point from protein abundance values. The outlier protein alongside three other proteomic biomarkers were identified as the best predictors. The class groups are coded high disease activity (class 0), secondary high disease activity (class 1), and low disease activity (class 2).

**Figure S7.** The distribution of samples over 12 mass spectrometry batches for the three collection time points and by disease activity status.

**Figure S8.** Missingness relationships and identification of the exemplar missingness outlier protein after adjustments for batch effect. A) The correlation between protein missingness separated by disease activity status. The magnitude of missingness for each protein identified in low disease activity participants (group 0) correlated with those measured in secondary high disease activity participants (group 1) to R=0.84, and to those measured in high disease activity participants (group 2) to R=0.81. The protein missingness measured in secondary high disease activity participants (group 1) correlated with that of low disease activity participants (group 2) to R=0.94. B-D) Identification of the outlier protein (highlighted in red) as a predictor disease activity status from missing values. The outlier protein is identified as an outlier due to increased missingness count in low disease activity participants when compared to both types of high disease activity participants. The protein’s missingness does not separate high disease activity from secondary high disease activity. The shaded area is a line parallel to the linear regression line, expanded in size.

**Figure S9.** The creation of reproducible missing values from standard proteomic bioinformatics. The images show the alteration in the magnitude of missing values in the exemplar protein. A) The protein has an increased magnitude of missing values after standard bioinformatic approaches over the collection time points where 0; baseline, 3; 3 months, 6; 6 months. B) The protein has increased missing-ness in patients with low disease activity and follows a similar trend regardless of bioinformatic quality control procedures. The disease activity groups are coded 0; high activity, 1; secondary high activity, and 2; low activity

**Figure S10.** The assessment of the distribution of missing values over 149 observations for proteins using different exclusion thresholds. A) The frequency of missing values for all identified proteins. B) The frequency of missing values for proteins with less than 30 missing values (<20% missing, >80% observations). C) The frequency of missing values for proteins with less than 50 missing values (>100 observations), as based on the plot distribution. D) The frequency of missing values for proteins with less than 75 missing values (<50% missing). E) The frequency of missing values for proteins with less than 134 missing values (<90% missing, >10% observations). The key below the figures shows the number of proteins remaining at each threshold for the corresponding plot.

**Figure S11.** A plot depicting the mean accuracy of the machine learning predictions at baseline and 3-months for the missing value thresholds. The figure shows the points for >10% observations (<90% missingness), <20% missingness (>80% observations), <50% missingness (>50% observations), all observations (0% missingness), and less than 50 missing values.

**Figure S12.** The assessment of missingness and proteomic predictions at different cut offs. The ‘Time’ column shows a correlation between protein missingness over three time points showed that the missingness count for proteins measured. The ‘Disease activity’ column shows a correlation between protein missingness separated by disease activity status. The ‘Baseline’ and ‘3 Months’ columns show the top two principal components in PCA plots depicting the prediction of level of disease activity by proteomic biomarkers at baseline and 3-month time points. The thresholds assessed alongside the 10% observations in the manuscript are; >50% observations, >80% observations, >50 observations, complete observations (0%). The class groups are coded high disease activity (class 0), secondary high disease activity (class 1), and low disease activity (class 2).

**>50%**: Baseline (group B) correlated with those measured at 3-months (group 3) to R=0.91, and with those measured at 6-months (group 6) to R=0.91. The protein missingness measured at 3-months (group 3) correlated with those measured at 6-months (group 6) to R=0.94. The missingness count for proteins identified in the high disease activity group (group 0) correlated with those measured in the secondary high disease activity group (group 1) to R=0.74, and to those measured in the low disease activity group (group 2) to R=0.70. The protein missingness measured in the secondary high disease activity group (group 1) correlated with that of the low disease activity group (group 2) to R=0.90. At baseline, 7 proteomic biomarkers predicted disease activity. At 3-months, 1 proteomic biomarker predicted disease activity and therefore there is no PCA plot.

**>80%**: Baseline (group B) correlated with those measured at 3-months (group 3) to R=0.80, and with those measured at 6-months (group 6) to R=0.84. The protein missingness measured at 3-months (group 3) correlated with those measured at 6-months (group 6) to R=0.86. The missingness count for proteins identified in the high disease activity group (group 0) correlated with those measured in the secondary high disease activity group (group 1) to R=0.63, and to those measured in the low disease activity group (group 2) to R=0.51. The protein missingness measured in the secondary high disease activity group (group 1) correlated with that of the low disease activity group (group 2) to R=0.77. At baseline, 9 proteomic biomarkers predicted disease activity status. At 3-months, 9 proteomic biomarkers predicted disease activity.

**>50:** Baseline (group B) correlated with those measured at 3-months (group 3) to R=0.84, and with those measured at 6-months (group 6) to R=0.87. The protein missingness measured at 3-months (group 3) correlated with those measured at 6-months (group 6) to R=0.89. The missingness count for proteins identified in the high disease activity group (group 0) correlated with those measured in the secondary high disease activity group (group 1) to R=0.68, and to those measured in the low disease activity group (group 2) to R=0.62. The protein missingness measured in the secondary disease activity group (group 1) correlated with that of the low disease activity group (group 2) to R=0.84. At baseline, 7 proteomic biomarkers predicted disease activity status. At 3-months, 24 proteomic biomarkers predicted disease activity.

**0%:** As there is no missingness, there is no plot of the Time and Disease activity columns. At baseline, 2 proteomic biomarkers predicted disease activity status. At 3-months, 10 proteomic biomarkers predicted disease activity.

**Supplementary Methods**

**Disease activity scoring**

Clinical measurements collected included swollen 28-joint count (SJC28), tender 28-joint count (TJC28), erythrocyte sedimentation rate (ESR) and patient global health assessment (PGA), as measured by visual analogue scale (VAS). Four component disease activity scores (DAS28-ESR) (0.56(√TJC28) + 0.28(√SJC28) + 0.70(ln(ESR)) + 0.014 × PGA) were calculated for each individual at 6-months. Where this was not possible, due to missing PGA in a small number of cases, a three component DAS28 was calculated as follows; [0.56(√TJC28) + 0.28(√SJC28) + 0.70(ln(ESR))] × 1.08 + 0.16.

**Sample preparation and proteomic analyses by SWATH**

Samples collected at each visit were stored at -80°C prior to analysis. Serum samples were prepared as described (Proc *et al.*, 2010) with the following adaptations. Major serum proteins were removed using Top 12 Abundant Protein Depletion Spin columns (Pierce Biotechnology, UK) and Amicon Ultra-0.5 Centrifugal Filter Devices (Merck-Millipore, UK) were used to concentrate the eluate and for buffer exchange. The depleted serum (normalised to 40 μg of protein per sample) was denatured, reduced, and alkylated in 25mM ammonium bicarbonate containing 5mM dithiothreitol (GE Healthcare, UK), 50 mM iodoacetamide (Sigma Aldrich, UK) and 1% sodium deoxycholate. Modified sequencing-grade trypsin (Promega, UK) was added and digestion performed overnight at 37°C. The samples were concentrated using a MiVac vacuum centrifuge GenevacTM (Thermo Fisher Scientific, UK).

Data independent acquisition (DIA)/SWATH-MS was performed using 100 variable precursor windows optimised for human serum. An ultra-high–performance liquid chromatography system (Eksigent ekspert nanoLC 400 autosampler and an Eksigent ekspert nanoLC 425 pump, AB SCIEX Ltd, UK) coupled to a SCIEX Triple TOF 6600 mass spectrometer with a DuoSpray Ion Source (AB SCIEX Ltd, UK) was employed. Samples were reconstituted in a buffer containing 5% (v/v) acetonitrile, 0.1% (v/v) formic acid, 100 fmol/μL of PepCalMix (MS Synthetic Peptide Calibration Kit, AB SCIEX Ltd, UK), and 10 x iRT (index retention time) standards (Biognosys AG, UK). After reconstitution, 10 μL of sample (containing 8 µg of total protein) was injected for chromatographic separation on a YMC-Triart C18 column (12nm, 150 x 0.3 mm) that was pre-coupled to a YMC-Triart C18 pre-column (12nm, 5 x 0.5 mm).

Reverse-phase chromatography was performed at 30°C with a flow rate of 5 μL/min over a 120-minute gradient. Mobile phase A contained 100% LC/MS water with 0.1% (v/v) formic acid and mobile phase B contained 100% acetonitrile with 0.1% (v/v) formic acid. Samples were run as duplicate injections with blanks between each sample. For SWATH-MS analysis samples were eluted with an analytical gradient (3 - 40% acetonitrile, 0.1% formic acid) and a mass spectrometry method with a total duty cycle of 2.8s comprising a TOF MS1 scan that was acquired over the mass range (m/z) 400 to 1250 followed by 100 SWATH-MS scans (m/z 100-1500) with variable m/z isolation widths, collision energy and collision energy spread. The voltage of spray was set at 5500V. Mass spectrometry-compatible K562 human protein extract (Promega, UK) was reconstituted as detailed for the samples and run as a technical control.

**Functional enrichment analysis**

Functional enrichment analysis of the identified outlier proteins (n=23) was conducted using DAVID (Huang *et al.*, 2009). The complete list of proteins identified in the serum samples (after a low threshold was removed) was used as the background for the analysis. All functional categories and protein domain databases were examined for significant enrichment within the outlier proteins.

Huang,D.W. *et al.* (2009) Bioinformatics enrichment tools: Paths toward the comprehensive functional analysis of large gene lists. *Nucleic Acids Res.*, **37**, 1–13.

Proc,J.L. *et al.* (2010) A quantitative study of the effects of chaotropic agents, surfactants, and solvents on the digestion efficiency of human plasma proteins by trypsin. *J. Proteome Res.*, **9**, 5422–5437.

**Supplementary Tables**

**Table S1**

Counts of missing values for each sample identified.

ID, sample identification; Timepoint, follow up time point of collection; DiseaseGroup, Disease status where 0 is high, 1 is secondary high and 2 is low disease activity; Count_Missing, the count of missing values out of the total 565 proteins identified above noise thresholds; Count_Proteins, the number of observations out of 565 proteins for each sample.

| ID | Timepoint | DiseaseGroup | Count_Missing | Count_Proteins |
| --- | --- | --- | --- | --- |
| 1 | BASELINE | 2 | 195 | 370 |
| 1 | 3 MONTHS | 2 | 188 | 377 |
| 2 | BASELINE | 2 | 170 | 395 |
| 2 | 3 MONTHS | 2 | 242 | 323 |
| 2 | 6 MONTHS | 2 | 199 | 366 |
| 5 | BASELINE | 2 | 218 | 347 |
| 5 | 3 MONTHS | 2 | 195 | 370 |
| 5 | 6 MONTHS | 2 | 206 | 359 |
| 6 | BASELINE | 2 | 173 | 392 |
| 6 | 3 MONTHS | 2 | 203 | 362 |
| 6 | 6 MONTHS | 2 | 216 | 349 |
| 7 | BASELINE | 1 | 181 | 384 |
| 7 | 3 MONTHS | 1 | 179 | 386 |
| 7 | 6 MONTHS | 1 | 191 | 374 |
| 8 | BASELINE | 1 | 196 | 369 |
| 8 | 3 MONTHS | 1 | 181 | 384 |
| 8 | 6 MONTHS | 1 | 172 | 393 |
| 9 | BASELINE | 1 | 210 | 355 |
| 9 | 3 MONTHS | 1 | 202 | 363 |
| 9 | 6 MONTHS | 1 | 174 | 391 |
| 10 | BASELINE | 0 | 187 | 378 |
| 10 | 3 MONTHS | 0 | 161 | 404 |
| 10 | 6 MONTHS | 0 | 184 | 381 |
| 11 | BASELINE | 1 | 179 | 386 |
| 11 | 6 MONTHS | 1 | 176 | 389 |
| 12 | BASELINE | 1 | 188 | 377 |
| 12 | 6 MONTHS | 1 | 169 | 396 |
| 15 | BASELINE | 2 | 220 | 345 |
| 16 | 3 MONTHS | 2 | 188 | 377 |
| 17 | BASELINE | 2 | 192 | 373 |
| 17 | 3 MONTHS | 2 | 190 | 375 |
| 18 | BASELINE | 2 | 190 | 375 |
| 18 | 3 MONTHS | 2 | 192 | 373 |
| 18 | 6 MONTHS | 2 | 238 | 327 |
| 19 | 3 MONTHS | 2 | 190 | 375 |
| 19 | 6 MONTHS | 2 | 136 | 429 |
| 20 | BASELINE | 1 | 186 | 379 |
| 20 | 3 MONTHS | 1 | 146 | 419 |
| 20 | 6 MONTHS | 1 | 191 | 374 |
| 21 | BASELINE | 2 | 158 | 407 |
| 21 | 3 MONTHS | 2 | 167 | 398 |
| 21 | 6 MONTHS | 2 | 152 | 413 |
| 22 | BASELINE | 1 | 136 | 429 |
| 22 | 3 MONTHS | 1 | 175 | 390 |
| 22 | 6 MONTHS | 1 | 134 | 431 |
| 25 | BASELINE | 2 | 123 | 442 |
| 25 | 6 MONTHS | 2 | 192 | 373 |
| 26 | BASELINE | 1 | 143 | 422 |
| 26 | 3 MONTHS | 1 | 124 | 441 |
| 26 | 6 MONTHS | 1 | 155 | 410 |
| 27 | BASELINE | 1 | 133 | 432 |
| 27 | 3 MONTHS | 1 | 168 | 397 |
| 27 | 6 MONTHS | 1 | 135 | 430 |
| 28 | BASELINE | 2 | 198 | 367 |
| 28 | 6 MONTHS | 2 | 190 | 375 |
| 29 | BASELINE | 1 | 183 | 382 |
| 29 | 3 MONTHS | 1 | 178 | 387 |
| 29 | 6 MONTHS | 1 | 136 | 429 |
| 30 | BASELINE | 0 | 208 | 357 |
| 30 | 3 MONTHS | 0 | 146 | 419 |
| 30 | 6 MONTHS | 0 | 190 | 375 |
| 31 | BASELINE | 2 | 162 | 403 |
| 31 | 3 MONTHS | 2 | 169 | 396 |
| 31 | 6 MONTHS | 2 | 170 | 395 |
| 32 | BASELINE | 0 | 169 | 396 |
| 33 | 3 MONTHS | 2 | 206 | 359 |
| 33 | 6 MONTHS | 2 | 205 | 360 |
| 35 | BASELINE | 1 | 113 | 452 |
| 35 | 3 MONTHS | 1 | 149 | 416 |
| 35 | 6 MONTHS | 1 | 163 | 402 |
| 36 | BASELINE | 1 | 186 | 379 |
| 37 | BASELINE | 2 | 178 | 387 |
| 38 | BASELINE | 2 | 180 | 385 |
| 39 | BASELINE | 2 | 200 | 365 |
| 39 | 3 MONTHS | 2 | 175 | 390 |
| 40 | BASELINE | 0 | 145 | 420 |
| 40 | 3 MONTHS | 0 | 145 | 420 |
| 40 | 6 MONTHS | 0 | 148 | 417 |
| 41 | BASELINE | 2 | 234 | 331 |
| 42 | BASELINE | 2 | 143 | 422 |
| 42 | 3 MONTHS | 2 | 147 | 418 |
| 43 | 3 MONTHS | 2 | 188 | 377 |
| 43 | 6 MONTHS | 2 | 200 | 365 |
| 45 | BASELINE | 0 | 159 | 406 |
| 46 | BASELINE | 2 | 195 | 370 |
| 46 | 6 MONTHS | 2 | 177 | 388 |
| 47 | BASELINE | 2 | 192 | 373 |
| 48 | BASELINE | 2 | 176 | 389 |
| 50 | BASELINE | 0 | 224 | 341 |
| 50 | 3 MONTHS | 0 | 212 | 353 |
| 50 | 6 MONTHS | 0 | 199 | 366 |
| 51 | BASELINE | 2 | 225 | 340 |
| 51 | 3 MONTHS | 2 | 230 | 335 |
| 51 | 6 MONTHS | 2 | 245 | 320 |
| 52 | BASELINE | 1 | 161 | 404 |
| 53 | BASELINE | 2 | 179 | 386 |
| 53 | 3 MONTHS | 2 | 187 | 378 |
| 53 | 6 MONTHS | 2 | 149 | 416 |
| 54 | BASELINE | 0 | 222 | 343 |
| 54 | 3 MONTHS | 0 | 155 | 410 |
| 54 | 6 MONTHS | 0 | 174 | 391 |
| 55 | BASELINE | 0 | 190 | 375 |
| 56 | BASELINE | 1 | 182 | 383 |
| 56 | 3 MONTHS | 1 | 172 | 393 |
| 60 | BASELINE | 1 | 257 | 308 |
| 61 | BASELINE | 1 | 157 | 408 |
| 61 | 3 MONTHS | 1 | 195 | 370 |
| 61 | 6 MONTHS | 1 | 233 | 332 |
| 62 | BASELINE | 2 | 183 | 382 |
| 62 | 3 MONTHS | 2 | 182 | 383 |
| 62 | 6 MONTHS | 2 | 214 | 351 |
| 63 | BASELINE | 0 | 180 | 385 |
| 63 | 3 MONTHS | 0 | 165 | 400 |
| 63 | 6 MONTHS | 0 | 165 | 400 |
| 64 | BASELINE | 1 | 194 | 371 |
| 64 | 3 MONTHS | 1 | 183 | 382 |
| 64 | 6 MONTHS | 1 | 186 | 379 |
| 65 | BASELINE | 2 | 160 | 405 |
| 65 | 3 MONTHS | 2 | 173 | 392 |
| 65 | 6 MONTHS | 2 | 170 | 395 |
| 66 | BASELINE | 2 | 218 | 347 |
| 66 | 3 MONTHS | 2 | 185 | 380 |
| 66 | 6 MONTHS | 2 | 155 | 410 |
| 73 | BASELINE | 1 | 243 | 322 |
| 73 | 3 MONTHS | 1 | 199 | 366 |
| 73 | 6 MONTHS | 1 | 208 | 357 |
| 74 | BASELINE | 2 | 162 | 403 |
| 74 | 3 MONTHS | 2 | 203 | 362 |
| 74 | 6 MONTHS | 2 | 238 | 327 |
| 75 | 3 MONTHS | 2 | 192 | 373 |
| 75 | 6 MONTHS | 2 | 200 | 365 |
| 76 | BASELINE | 0 | 186 | 379 |
| 76 | 3 MONTHS | 0 | 211 | 354 |
| 79 | BASELINE | 0 | 188 | 377 |
| 79 | 3 MONTHS | 0 | 190 | 375 |
| 79 | 6 MONTHS | 0 | 228 | 337 |
| 80 | BASELINE | 0 | 178 | 387 |
| 80 | 3 MONTHS | 0 | 158 | 407 |
| 81 | BASELINE | 2 | 221 | 344 |
| 81 | 3 MONTHS | 2 | 237 | 328 |
| 81 | 6 MONTHS | 2 | 223 | 342 |
| 82 | BASELINE | 2 | 193 | 372 |
| 82 | 3 MONTHS | 2 | 206 | 359 |
| 82 | 6 MONTHS | 2 | 194 | 371 |
| 83 | BASELINE | 1 | 201 | 364 |
| 83 | 3 MONTHS | 1 | 204 | 361 |
| 83 | 6 MONTHS | 1 | 184 | 381 |
| 84 | 3 MONTHS | 1 | 200 | 365 |
| 84 | 6 MONTHS | 1 | 192 | 373 |
